# Institutionalizing Experimental Places for Inclusive Social Innovation: From Utopias to Heterotopias

**DOI:** 10.1007/s11266-023-00584-w

**Published:** 2023-06-12

**Authors:** Alessandro Sancino, Fulvio Scognamiglio, Luigi Corvo, Francesca Imperiale, Giulio Pasi

**Affiliations:** 1 The Open University, Milton Keynes, UK; 2 University of Milan-Bicocca, Milan, Italy; 3 University of Salento, Lecce, Italy; 4 Universidad Loyola, Seville, Spain

**Keywords:** Social innovation, Local government, Civil society, Organization studies, Welfare state

## Abstract

This essay embraces a notion of critical scholarship concerned with proposing normative and actionable alternatives that can create more inclusive societies and focuses on the role of institutionalizing experimental places for inclusive social innovation as a bottom-up strategic response to welfare state reforms. By mobilizing the notions of utopias and heterotopias in Foucault, the paper sheds light on the opportunity to move from policy utopias to democratic heterotopias, discussing the politics embedded in this cognitive shift and the democratic nature of social innovation changing social and governance relations by interacting with politico-administrative systems. Some obstacles to institutionalizing social innovation are highlighted, as well as some key governance mechanisms that can be activated either by public and/or social purpose organizations to try to overcome those obstacles. Finally, we discuss the importance of linking inclusive social innovation with democratic, rather than market logics.

## Introduction

Our society is suffering from increasingly complex and entrenched challenges (George et al., 2016), such as for example climate change, food insecurity, freedom and human rights, international migration, and socio-economic inequalities. A specific category of these problems, or even a consequence of the interweaving of them, is the welfare state crisis with the related reforms that have happened in the last 50 years in the attempt to deal with it (e.g., Baglioni, 2017; Nicholls et al., 2015). Indeed, after the 1970s with the increased demand and the widening of the variety of social needs from the population and specific demographic changes,^1^ there has been a huge amount of pressure and demands on national and supranational entities in reforming the welfare state and in the provision of existing social services (e.g., Pestoff, 2012). One solution provided by several countries has been to decentralize the welfare state while simultaneously recurring to the market for the provision of a wide array of social services (e.g., Henriksen et al., 2012). However, these New Public Management (NPM) reforms have in many cases weakened the capacity of civil society and the public sector to deal with wicked issues, especially in terms of not only delivering, but also innovating and finding new social relations and solutions, with the effect of often generating losses of perceived legitimacy of government action by the citizens (Dey &Teasdale, 2016).

Against this backdrop, this essay starts with a relevant and urgent problem (Carboni et al., 2019), namely the reform of the welfare state to deal with increasingly complex social protection needs, and embraces a notion of critical scholarship as concerned with proposing normative and actionable alternatives that can create more sustainable societies (Coule et al., 2022). Specifically, we advance the idea that institutionalizing and connecting experimental places for inclusive social innovation can be a bottom-up strategic response to the welfare state crisis, specifically a democratizing response that conceives social innovation as an inclusive and collective practice of democratic learning and deliberation with marginalized citizens and relevant publics, rather than merely being a practice led by empowered and heroic actors.

Engaging with debates occurring in this journal on the emancipatory potential of social innovation beyond neo-liberal governmentalities (Lachapelle, 2021) and on the technocratic vs. democratic approach to social innovation (Montgomery, 2016), we follow a critical performative stance (Alvesson & Spicer, 2012) where through a tactic of progressive pragmatism we engage with present potentialities ‘to create a sense of what could be’ (Alvesson & Spicer, 2012, p. 377). Inspired by Lachapelle (2021) and Mazzei et al. (2021), we illustrate how institutionalizing and connecting places for inclusive social innovation requires a move from utopias to everyday civic opportunities for allowing different publics to experience social innovation. By mobilizing the notion of utopias and heterotopias in Foucault, we shed light on the opportunity to move from policy utopias to democratic heterotopias discussing the politics embedded in this cognitive change and bringing up, as recently pointed out by Mair et al. (2023) and by Battilana and Casciaro (2021), the political nature of social innovation. The notion of heterotopias is important to make a cognitive shift where social innovation is not anchored in binary paradigms, but co-existing with other governmentalities and paradigms emerging from post-capitalistic and neo-democratic features (Zanoni et al., 2017), and potentially creating new hybrid and democratic proto institutions (e.g., Lawrence et al., 2002; Skelcher et al., 2013).

Our proposed alternative is that of a partnership between the state and civil society and third sector (Macmillan, 2020), with social innovation becoming a problem oriented permanent operational programme located in a hybrid space characterized by ‘complex interactions that cut across sectors’ (Dekker & Evers, 2009, p. 219).

The idea for this essay came from conversations among the co-authors (comprising an international team of scholars engaged in the social innovation literature and one professional policy expert) about the actual state of social innovation institutions, the actual barriers to democratic institutionalization and the possible instruments to enact. The result of these discussions has been a collective essay of critical scholarship about social innovation inclusiveness and institutionalization that combines the theoretical knowledge developed by the academic side of the authors and the key insights stemming from the challenges, successes, and backlashes experienced by the policy expert over many years devoted to design and implement effective policy measures to make social innovation flourishing in the European Union institutions. Therefore, in the spirit of increased exchange of ideas between policy and academia, this collective essay is structured as follows.

The next section provides a conceptual backdrop where we define some key terms, such as institutionalization, experimental places, publics, utopia and heterotopia as well as our positional approach and perspective to social innovation. After having clarified our *thesis*/position, methodologically we proceed with an *antithesis* and *synthesis* approach grounded in the Greek philosophical tradition, so the third section discusses some obstacles that may hamper the institutionalization of experimental spaces for inclusive social innovation (*antithesis*), while the fourth section, symmetrically, highlights several mechanisms for overcoming these obstacles (*synthesis*). Finally, the last section provides some concluding remarks where we also discuss some managerial implications for public and social purpose organizations in terms of moving from policy utopias to democratic heterotopias.

## Theoretical Backdrop: Institutionalizing Social Innovation Between Utopias and Heterotopias

The issue of if and how institutionalize social innovation is not new and has generated a rich debate (e.g., Brandsen et al., 2017). Drawing from Hjelmar (2021, p. 55) ‘institutionalization is viewed as the process in which assumed ideas are transformed into operational programmes that are accepted as effective ways to achieve the agreed objectives’; as written by Agostini et al., (2017, p. 389) citing Cajaiba-Santana (2014) ‘the institutional perspective sees social innovation as a result of the exchanges and application of knowledge and resources by agents mobilized through legitimization activities.’

According to the TRANSIT (“TRANSIT—TRANSformative Social Innovation Theory”) research project,^2^ institutionalizing social innovation deals with how ‘social innovation as perceived by key governance actors and its associated practices is institutionalized, either anchored through newly established institutions or embedded in existing ones’ (Pel & Bauler, 2014, p. 2). This stream of research focused explicitly on the institutionalization of social innovation and defined Transformative Social Innovation as ‘social innovation that challenges, alters or replaces dominant institutions in the social context’ (Avelino et al., 2019, p. 206). Other scholars, such as for example van Wijk et al. in a special issue in business and society, have pointed out that ‘innovations are able to address a persistent problem only insofar as they gain permanence through their institutional embedding (van Wijk et al., 2019, p. 890).’

Clearly, being social innovation an umbrella concept and a “deeply political boundary object” (Pel & Bauler, 2014), it is important to clarify the position and perspective taken to social innovation. Here, we consider social innovation as ‘new ways of doing (practices, technologies, material commitments), organizing (rules, decision-making, modes of governance), framing (meaning, visions, imaginaries, discursive commitments) and knowing (cognitive resources, competence, learning, appraisal)’ (Pel et al., 2020, p. 3)’ that occurs ‘through an open process of participation, exchange and collaboration with relevant stakeholders, including end users, thereby crossing organizational boundaries and jurisdictions’ (Voorberg et al., 2015, p. 1334). According to Marques et al., (2018, p. 501), there are at least four meanings of social innovation: (i) structural social innovation which results in a wide socio-economic-political change; (ii) targeted radical social innovation which radically reshapes how goods and services are delivered and power relations; (iii) targeted complementary social innovation which generate new processes and relationships for inclusive solutions to social challenges; (iv) instrumental social innovation which is basically about rebranding of existing practices/endeavours.

Overall, like Agostini et al., (2017, p. 385) we take a pragmatic view of social innovation as ‘a way to mitigate social problems, resulting in new or improved solution for a specific community’ which should generate new social and governance relations. Indeed, social innovation can be considered as such if ‘contribute to changing social relations’ (Pel et al., 2020, p. 3) and governance relations that ‘include the interaction with and the embedding into the politico-administrative system of the democratic states of the countries to which the communities belong (Moulaert et al., 2005, p. 1973, cited in Tello-Rozas, 2016).’ In this paper, we take a mixed position between the targeted radical and complementary view of social innovation, but also considering the structural social innovation with a bottom-up approach that aims to produce structural social innovation by way of accumulation of institutionalized and connected experimental practices/endeavours that change social and governance relations interacting with the politico-administrative systems.

This perspective to social innovation brings in especially the role of public sector institutions and civil society in experimenting with social innovations. This is usually a neglected focus, especially from scholars within organization and management studies with a main focus on the commercial sector, which has the merit of overcoming a rigid separation between state, market and civil society (Domanski et al., 2020). Most importantly, it connects with recent developments and debates on social innovation and democracy and on collaborative forms of open social innovation (e.g., Mair & Gegenhuber, 2021). To this regard, the notion of publics we believe is particularly important here and we introduce it now.

Publics are ‘forums gathering together groups of citizens to deliberate on a particular issue…where participants are recruited through specific methods that ensure the representation of different societal groups or viewpoints’ (Setälä, 2017, p. 846). According to Fung, a publics ‘is made up of citizens who come together to reflect upon collective affairs and state actions in to jointly discover and create new, more effective approaches and possibilities’ (Fung, 2002, p. 68).

Thus, designing, institutionalizing, and connecting experimental places for inclusive social innovation with publics and relevant stakeholders becomes a practice aimed at understanding social innovation from a democratic learning and civic capabilities development point of view (Sancino, 2016), rather from the perspective of profitization through market logics. This perspective questions also the idea of “scaling up” as superior form of social innovation as opposed to “scaling out” (see on this for example Westley et al. 2014, p. 236 and Zuckerman, 2020), where scaling—in any of its forms—should be assessed against a “philosophy of evolutionary learning”(e.g. Ansell, 2011)’.

The focus on interactions between public sector institutions and interactions with publics brings in ideas around policy utopias (Mazzei et al., 2021) and instrumental/complementary social innovation for keeping public services running through co-production within a regime of austerity. For example, Mazzei et al. (2021, p. 1636) have illustrated how ‘SE in the UK policy context has been mobilized as a superior model of social provision’ that may result ‘in the discursive construction of a utopian programme ‘consigned to the role of a ‘wish-fantasy’ (Levitas, 1979), rather than a catalyst of hope and, possibly, change’ (pp. 1637–1638).

Mobilizing Foucault (Vidler et al., 2014), we embrace here a distinction between utopias and heterotopias. Utopias, as Foucault explained, are imaginary places that represent an ideal or perfect society. They are often meant to inspire the creation of a better society, but never feasible, with the main function to provide an ideal image of society. Symmetrically, heterotopias are real spaces that have the capacity to contain different meanings and functions simultaneously. Foucault cites examples such as museums, prisons, theatres, and gardens as examples of heterotopias. These places are spaces where the rules and functions of society are suspended or mixed in a way that does not happen elsewhere, creating a sort of "elsewhere" in our world. If we think about social innovation initiatives, it is tellingly how the examples of heterotopias just made have been often fertile grounds for the spread of myriad of social innovations (e.g., restaurants run by incarcerated people, cultural museums owned by local population, theatral performances as cultural sites for democratic mobilization, urban gardens, etc.).

Accordingly, the concept of institutionalizing and connecting heterotopias as experimental places for inclusive social innovation seems particularly important for answering at demands of concreteness against utopian expectations (Pel & Bauler, 2014), because virtually any public purpose organizations could activate heterotopia places even within existing institutional arrangements. Clearly, there are issues in terms of the redistribution of power among the actors involved to be considered, but at least the moving from a utopian to a heterotopia imaginary gives back the power for agency (Battilana & Casciaro, 2021) to all the public purpose organizations to work concretely with different publics to develop social innovation capacity. In the next section, we illustrate some obstacles and some mechanisms to foster this transition.

## Institutionalizing Social Innovation as a Response to Welfare State Crisis: Several Obstacles

### Social Innovation as Entrepreneurial Micro Problem-Solving

Despite a growing interest for social innovation initiatives, which have seen an increasing amount of available funding from national and supranational governmental bodies, the idea of social innovation as an attractive or even pursuable way of dealing with the broader rethinking of the welfare state (Ewert & Evers, 2014), and more generally as a practice of democratic active citizenship, has not often being considered. Indeed, policymakers are often more interested in economic approaches to deal with the social needs of the different societal stakeholders. The evidence is in the wave of NPM reforms from the 1980s that rolled over several national welfare systems by recurring to the privatization and/or the marketization of providing social services.

A certain notion of social innovation, mainly shaped by the pragmatic and managerial schools (e.g. Mulgan et al., 2007; Phills et al., 2008), has prevailed among the many available, such as for example the systemic and critical ones (e.g. Moulaert et al., 2005; Westley et al., 2017). Indeed, the definitional focus on the micro problem-solving and presentist nature of social innovation has shed a shadow on other and potentially more system-change features of social innovation (Busacca, 2013), such as the process of “empowerment and political mobilisation” (e.g. Moulaert et al., 2005) and the role of social innovation in building resilience through transitions (e.g., Westley et al., 2017).

### The Embeddedness Paradox: Fighting Their Own Institutions

Another challenge on the institutionalization journey is the embeddedness paradox inherent to the social innovation approach, characterized by a combination of path discontinuity and path dependency from the existing institutions (Avelino et al., 2019). As we have said above, social innovation approaches are being designed and implemented often because of the crisis that the welfare state is experiencing and of the difficulty in providing the social needs that citizens and other societal stakeholders ask for (Parthasarathy et al., 2021). The innovation side of the social innovation approach is intended to innovate existing practices that do not fulfil their promises. This means that social innovation initiatives aim to fill a void left by the existing institutions by trying to implement a new and discontinue approach from the past (Cajaiba-Santana, 2014).

However, we must take into consideration that any social innovation initiative does not happen in an institutional vacuum, but it is rather surrounded by a universe of already present norms and practices (Pel et al., 2019). Thus, existing institutions may act as exogenous enabling factors that prevent the possible paths of social innovation initiatives (Jensen & Fersch, 2019). These factors could be budget restraints, constitutional norms, or place-based constraints of various natures such as a culture adverse to collaboration, high power distance, low civic capacity, and more broadly diverse relational assets (Mazzei, 2017). In addition to that, existing institutions may try to resist from the path discontinuity initiatives that social innovation approaches try to implement.

### Public Institutions Paradox: A New Role?

Another barrier to the thriving of social innovation approach as an institutional way to deal with welfare state reforms is how and what presence is needed from the public sector institutions. Indeed, as we have said before, a social innovation approach is usually taken to provide social needs that have been unmet by the existing welfare state (Phillips et al., 2015). So, a lack of public sector actor action is a fundamental trigger for the rise of social innovation initiatives, reconnecting to the path discontinuity approach explained above. However, social innovation may need some resources from the public institutions, ranging from financial instruments to facilitating environment in which this approach could take form. So, too much lack of public institutions presence could severely affect the outcomes and a successful impact of social innovation initiatives. This is why public and social purpose organizations could open up spaces and redesign themselves and the environment around them in order to help by facilitating social innovation to satisfy those unmet social needs (Austin, 2003; Lenz & Shier, 2021).

### Social Innovation: A Difficult Definition and a Plethora of Perspectives

Another barrier on the way to institutionalize social innovation as a strategic approach to deal with the wickedness of welfare state crisis is the difficulty on defining it (Solis-Navarrete et al., 2021). When public and social purpose organizations are attempting at implementing social innovation initiatives, they often do not have a clear definition in mind, undermining the possibility of success and impact of them. Indeed, while heterotopias could be considered as constructive discursive places in which different perspectives should thrive and coexist, an excess of conceptual ambiguity could trigger a deep ontological crisis of social innovation and its institutions (Gruber, 1995). Drawing from the social innovation literature, we have been able to identify three definitional challenges through which the concept has passed and is still dealing with (Marques et al., 2018; Ayob et al., 2016).

The first ambiguous element of the definition is in what could be defined innovation and what not (Baptista et al., 2019). To this regard, an initiative could be considered innovative if it develops a completely new approach or implement an innovative solution developed in another context through a process of adaptation. In this respect, while commercial innovation enjoys some regulatory protection at global scale, this does not exist for social innovation. Also, while commercial innovation is often realized once for all (kind of accumulation process), this does not apply to social innovation (Teasdale, 2012). This peculiar nature of social innovation shows that social innovation is extremely context-dependent: what in a given space could be considered a social innovation does not necessarily need to be in another context (Moulaert & MacCallum, 2019; Van Dyck & Van Den Broeck, 2013; Moulaert et al., 2009).

The second ambiguous element is the social character of these initiatives. Indeed, the innovation concept has been traditionally connected to the market sector with the aim of producing profit for the stakeholders involved. However, social innovation initiatives, regardless of the different approaches taken, are usually implemented to satisfy unmet social needs not necessarily related to monetary or financial aspects (Nicholls et al., 2015). This aspect does not imply that social innovation initiatives should not take into consideration financial sustainability. Indeed, when stakeholders aim for a long-term impactful social innovation initiative, they have to make compatible the social and the financial aspect of their approach (Mazzei et al., 2021; Teasdale, 2012). Nevertheless, they must not overlook on the fact that their main goal is to provide new solutions to social problems and not just make profit with an innovative solution.

A third element at the base of social innovation definitional ambiguity is the conflation with other innovation approaches (Solis-Navarrete et al., 2021). Indeed, as said above, social innovation is a specific approach with specific goals that must be precisely distinguished from others. There are several examples of what social innovation is not: traditional innovations that are strictly business-oriented; social entrepreneurship which often lacks of scaling up capabilities and involvement of actors outside the business sector; frugal innovations which are more oriented to reducing the cost of a specific product or process; or environmental innovations which regardless of their social impact are mostly implemented to deal with economic issues.

### Coordinating Actors: Different Perceptions and Different Governance Games

Another fundamental challenge to design, implement, or simply facilitate a social innovation approach stands in the difficulty of coordinating different societal stakeholders. Indeed, wicked problems need wicked adaptations, such as a social innovation response (Zivkovic, 2018). However, these kinds of adaptations require the involvement of all the publics and stakeholders that are affected by the problem, which in the case of the welfare state crisis and attempts to reform it are all the sectors of society (Misuraca et al., 2018). Indeed, the difficulties in dealing with wicked problems impede a single actor from solely dealing with them and ask for the pooling of the resources of a Quadruple/Quintuple Helix model of actors’ involvement, which is the production of knowledge and innovation with the participation of organized stakeholders coming from all the different societal sectors (Carayannis et al., 2018), as well as the relevant publics of citizens which already exist or might come to exist because of called into being by a convening act (Moulaert et al., 2017; Sancino et al., 2021).

From the necessary involvement of all these different actors follows a predictable difficulty in coordinating them. Indeed, the lack of capability from the actor that promotes a social innovation approach in dealing with such a wide array of actors could lead to either the exclusion of some of the societal stakeholders with all the consequences in loss of efficacy and legitimacy or the derailing of the social innovation to an inevitable failure. Drawing from the network governance literature, we have identified three interrelated coordination challenges that affects social innovation institutions (Klijn & Koppenjan, 2016).

The first coordination challenge concerns the difference in perceptions of the different publics and stakeholders (Avelino et al., 2019). Indeed, the necessity of involving actors coming from different sectors of society results in a plethora on different perspectives on how to design, how to implement, what are the instruments or the goals that a social innovation initiative must have. It is not unusual that social innovation initiatives spend a lot of resources in managing the different actors’ perceptions, with also the risk of failing before implementing due to a lack of shared intentions.

The second coordination challenge lies in the difficulty of managing different publics and stakeholders on a daily basis (Källström et al., 2021; Nicholls et al., 2015). Indeed, a social innovation initiative is a difficult strategic game that could not be planned in advance due to frequent upheavals. The third coordination challenge has been already analysed above and concerns the institutional complexity in which social innovation initiatives take place (Ewert & Evers, 2014). Indeed, as we have already observed, the clash between existing norms and routines and new institutional settings proposed by social innovation calls for a necessary institutional managing of this transition (Borzaga & Bodini, 2014). Indeed, recognizing that social innovation initiatives are usually implemented at the expenses of specific actors implies a whole set of consideration on how to set a self-sufficient subsequent institutional equilibrium that does not punish the old institutional actors.

### Challenges in Measuring an Ambiguous Concept

Social innovation as a concept has been traditionally afflicted by the problem on how to measure the impact on society (Unceta et al., 2016). In particular, the road to institutionalize such an approach necessarily passes through the evidence that these initiatives are capable of achieving their goals of fulfilling unmet social needs and have a significant impact on the society.

The first problem for a more institutionalized measurement system is in the struggle of defining what is social innovation and what are its goals (Pel et al., 2019). Indeed, the definitional divergence due to the ambiguity of the concept but also the involvement of different publics and stakeholders with often conflicting perspectives represents a key obstacle for policymakers on finding a common measurement approach.

The second challenge is represented by an objective and subjective lack of data (Mihci, 2020). It is not unusual that social innovation initiatives impact lack of a serious and well-defined measurement system with the consequence of an absence of reliable sources of data both for researchers but also for policymakers.

The third challenge faced in defining a measurement system for the social innovation is at which level we should measure the impact of social innovation initiatives (Dyck & Broeck, 2013). Indeed, a long-standing debate within the social innovation community is to decide what is the most appropriate level at which set the indicators and measure the impact and the outcomes of a social innovation initiatives (Mihci, 2020).

## Institutionalizing Experimental Spaces for Inclusive Social Innovation: Several Governance Mechanisms

### Widening Through Information Sharing and Co-creation

The path for the institutionalization of social innovation as a heterotopic concept must pass through a purposeful widening of the actors involved into social innovation initiatives. Indeed, while specialization by academics and practitioners in both theoretical and empirical aspects of social innovation has been necessary for the foundation of the concept, now it might still have a scarce acquaintance outside specialists of what is social innovation and its value. What we propose here is a combination of reactive and proactive involvement of publics in a renovated, heterotopic social innovation through information sharing from specialists and the involvement of a variety of societal stakeholders through open social innovation approaches.

The information sharing approach consists into the popularization of social innovation knowledge beyond the usual places. Indeed, a more proactive approach from stakeholders, lay actors and ordinary citizens must pass through the acknowledging of both existence and importance of social innovation from a multitude of publics. For example, academics and practitioners could include a necessary activity of dissemination and engagement with the educational system, starting from the early stages in school to transform social innovation as a familiar institution for citizens. Secondly, public and social purpose organizations could develop specific knowledge creating organizations for social innovation education. These activities will lay the foundation for a citizens’ empowerment in the social innovation institutionalization to deal with the welfare crisis, framing them not only as users or consumers of services but rather as civic and community actors caring for an increasing relational welfare.

The second widening approach that public and social purposes organizations could undertake is inspired by the Open Social Innovation conceptual framework (e.g., Chesbrough & Di Minin, 2014). A plausible governance mechanism for public and social purpose organizations to involve a wider range of publics is through co-creation processes, with a distributed rather than single agency driven interaction and with the participation of lay rather than only organized actors and with a focus on innovation rather than merely on alignment (Sørensen & Torfing, 2019; Torfing et al., 2019).

### Layering and Redesigning for Institutionalization

As we have seen above, the institutionalization of social innovation puts potentially an important load on the actors coming from the public sector. In this regard, the two paradoxes outlined in “The Embeddedness paradox: Fighting Their Own Institutions” and “Public Institutions Paradox: A New Role?” sections could be intertwined generating two fundamental challenges for the public sector in dealing with the institutionalization of social innovation. The path discontinuity highlighted as one side of the embeddedness paradox goes hand in hand with the lack of public sector action as a trigger for social innovation; moreover, the path dependency from the institutional context in which social innovation institutionalization takes place could be coupled to the necessary number of resources provided by the public sector institutions to activate social innovation initiatives.

However, a possibility of contemporary dealing with both these two obstacles could come from the capability of the public sector actor to combine different institutional logics and achieve an institutional layering equilibrium, namely a public sector capable of transforming its institutions by adding new capabilities through an incremental evolution process (Hartley, 2005; Mahoney & Thelen, 2010). What we propose here is an observation of what is already ongoing in the public sector, namely the awareness from the public sector of the unsustainability of its traditional governance and institutions in the fiscal and welfare crisis of the last three decades. These collective problems are too multifaceted and complex to be solved by a public administration built on the industrial paradigm of the machine (hence the metaphors of the administrative machine or bureaucratic machine).

Public administration systems were indeed built to give (standardized) answers implemented by hierarchical mechanisms to a world now grappling with complex and wicked challenges. Thus, increasingly, the new collective social problems are now dealt with by the intervention of multiple actors (profit, non-profit, or social entrepreneurs). The examples are endless and cover many issues: car sharing, neighbourhood social networks, corporate welfare, garbage collection, commons care, co-working, etc. Embracing a multi-actor perspective (e.g., Bryson et al., 2014) means putting the welfare of communities at the centre and starting from there to organize the functioning of public and social value chains. It means putting at the centre the public and social value missions (e.g., Mazzucato, 2021) and calling stakeholders and publics to contribute; it means that at the heart of collective action, there are the problems/aspirations of the community and their solutions.

In addition to a more mission-oriented public administration, we highlight the central role that municipalities are taking and should take into the institutionalization of the social innovation approach (Brandsen et al., 2016). Indeed, municipalities, as the democratic pivot of place-based governance, can institutionalize experimental places for social innovation initiatives, considering experimentation as a fundamental value of pragmatism and democratic experimentalism as a distributed learning architecture (Dorf & Sabel, 1998).

Municipalities are the most context-aware level among the public sector institutions, helping the layering transition dealing with the path dependency of existing institutions. Indeed, in their relation with the other societal stakeholders and publics for innovation purposes, local authorities could cover a wide range of role, ranging from the network formation and collaboration, narrative construction and communication, bridging agency (Lenz & Shier, 2021) and/or promoter, enabler or partner of innovative arrangements (Kronsell & Mukhtar-Landgren, 2018). Municipal meta-governance (Sørensen, 2006) may be important for social innovation initiatives which often require public support to being enabled and to flourish. A concrete example is the “Citizen’s Agreement for an Inclusive Barcelona”, a social innovation proposed by the municipality that promotes the creation of several urban independent networks of different societal stakeholders and publics with the common aim of developing inclusive social welfare policies (Montagut et al., 2016).

### A convener and a Facilitator: Two Different Capabilities

As we have underlined in the “Social Innovation as Entrepreneurial Micro Problem-Solving” and “Social Innovation: a Difficult Definition and a Plethora of Perspectives” sections, the institutionalization of social innovation as a heterotopia passes through the challenges of avoiding an ontological crisis of the concept and coordinating possibly conflicting actors involved in the institutionalization processes. What we suggest in this sub-section is a set of governance tools and mechanisms that could help public and social purpose organizations to acquire new capabilities and be ready to successfully implement social innovation approaches for welfare state reforms. Creating a favourable environment for the societal stakeholders to design, discuss, implement, and measure social innovation initiatives requires a wide array of capabilities and governance tools to be combined in a creative way (Pel et al., 2020).

The first issue that we deal with is the convener role, namely the capacity of bringing together all the actors that are considered fundamental to make social innovation initiatives successful (e.g., Nicholls et al., 2015; Sancino, 2022). Indeed, as we have underlined in the section dedicated to the layering institutionalization, complex solutions as a social innovation approach require a vast range of resources that any actor solely possesses. Public and social purpose organisations must be able to identify the necessary resources to deal with the problems spilling from the welfare crisis and be able to convene those actors possessing these resources (Kania et al., 2014). Another fundamental issue for conveners is to make the stakeholders and publics involved aware of their mutual resource dependency (Roy et al., 2014). This explaining role must be implemented at the beginning of social innovation initiatives for its propaedeutic nature for the future collaboration and to build mutual trust relationships among all the stakeholders involved into the process.

Besides the involvement and the bounding of actors, public and social purpose organizations should use a series of governance mechanisms to make the stakeholders and publics involved collaborating. Without claiming to be exhaustive, what follows are several examples of governance mechanisms that have proved to be successful in social innovation practices and that public and social purpose organizations could recur to by adapting to their specific social innovation approach. The first governance mechanism concerns the reinforcement of existing partnerships. Indeed, building new collaborations among stakeholders unknown to each other is often an expensive and time-consuming activity. If and when present, public and social purpose organizations should take advantage of the existing trust between stakeholders, a fundamental resource to deal with conflicts and tensions arising while tackling wicked problems. This is especially relevant when the contextual conditions change abruptly and there is necessity of recurring to existing long-term partnerships to concentrate relevant resources for dealing with external problems (Pel et al., 2019). However, this mechanism must be used with particular attention due to the risk of reproducing existing institutions and act as gatekeepers towards new but relevant stakeholders (Ruddat & Schönauer, 2014).

Another particular governance mechanism is emotional responses from stakeholders and publics (e.g., Garcia-Orosa & Pérez-Seijo, 2020). Indeed, the proactivity and the willingness to collaborate with other actors into social innovation processes are expected to be higher when stakeholders are not only rationally, but also emotionally involved in the issue that is dealt with. An example could be during COVID-19 where multi-stakeholders social innovation processes happened in unexpectedly rapid and consistent ways due to the turbulence of the pandemic (e.g., Ansell et al., 2021). This is not a governance mechanism appliable for every social innovation process so it must be implemented only within specific contexts.

As we have seen in different sections above, definition issues in social innovation processes cover a fundamental position. At this regard, public and social purpose organizations could have an important facilitating role when the perceptions conflicts arise to avoid that social innovation processes end up in a quagmire (Avelino et al., 2019). Indeed, as facilitators, they should set some knowledge boundaries acceptable for all the stakeholders involved and where they could discuss about the direction of the social innovation process (Baptista et al., 2019). These knowledge boundaries represent the common ground in which stakeholders must remain to not compromise the social mission of the process. In summary, the capability of public and social purpose organizations to establish a sustainable discourse formation and its sustainability is a fundamental prerequisite for the success of any social innovation initiative.

### Drawing Boundaries and Measuring Social Innovation

The different barriers that challenge social innovation as both an approach and as an institution affect the possibility of measuring its outputs, outcomes, and impact, as we have seen in the “Coordinating Actors: Different Perceptions and Different Governance Games” section. This is an issue common to all magic concepts or buzzwords, such as sustainability (Bock, 2012), circular economy (Friant et al., 2020), or sharing economy (Henry et al., 2021). In this regard, public and social purpose organisations have at their disposal several mechanisms.

A preliminary step is to clarify what are the theoretical and empirical boundaries of social innovation. Indeed, to deal with the challenges spilling from a variety of perspectives within a heterotopic social innovation, public and social purpose organizations must acquire and improve their ability to recognize social innovation practices that are implemented only for window dressing purposes and/or for profit maximization. Window dressing is becoming increasingly frequent, with several programmes often masked as social innovation practices towards “efficiency” or “freedom of choice” of citizens treated as customer (Baptista et al., 2019). These initiatives are often only a revival of already failed NPM reforms which try to get a second life by dressing it as social innovation practices. One of the consequences of this phenomenon is the profit maximization that certain stakeholders involved into social innovation practices aspire to. Indeed, we must distinguish traditional economic innovations, which have profit as their final goal, and social innovation initiatives that try to satisfy the unmet social needs that are often the result of efficiency-seeking and profit-safeguard marketization and privatization welfare state reforms.

However, distinguishing social innovation from other kind of approaches does not equate with a clarified measurement system. One of the main challenges for a measurement system is the objective and subjective lack of high-quality data (Ewert & Evers, 2014). Indeed, as we have seen in the “Coordinating Actors: Different Perceptions and Different Governance Games” section, the road for the implementation of an assessment and measurement system is paved with a multitude of challenges (Mihci, 2020). In this regard, a sustainable and open for lesson drawing measurement system must be created to be open and easily accessible not only to specialists but also to the widened spectrum of lay actors involved, following the approach behind the mechanism “Widening through Information sharing and Co-creation” section.

To this end, policymakers have different resources to implement an information system that could support social innovators. Examples are integrated electronic databases to avoid the fragmentation of data collection and storage. These databases could also be coupled with search services and malleable platforms by taking advantage of the platformization of our society (e.g., Ansell & Miura, 2020). In an individualized and network society, the platform can indeed become the dominant paradigm for a modern open co-governance (Meijer et al., 2019) of welfare, where technology is a key resource as are cooperation, trust and relationships. Platforms can foster pooling between demand and supply of welfare services, with tools such as marketplaces, including digital ones, that may encourage both the aggregation of demand as much as the qualification of supply in an integrated system of open co-governance of "community portfolios", overcoming the rigidities of the current system of supply bureaucratic "silos" that are not communicating (IFEL/ANCI-Cariplo, 2022).

In addition to that, an open measurement system must be known to be useful, with the necessity for policymakers of advertising the existence of such services, employing a network of information brokers, maintaining them by adjourning it frequently, and making them open to feedback from citizens to guarantee their usefulness (Table 1).

**Table 1:** A Summary of the barriers and the related mechanisms identified

Barriers	Mechanisms
3.1 Low interest for Social Innovation	4.1. Widening through Information Sharing and Co-Creation
3.2 The embeddedness paradox: fighting their own institutions 3.3 Public institutions paradox: a new role?	4.2. Layering and redesigning for institutionalization
3.4 Social Innovation: a difficult definition and a plethora of perspectives 3.5 Coordinating actors: different perceptions and different governance games	4.3. A convener and a facilitator: two different capabilities
3.6 Challenges in measuring an ambiguous concept	4.4. Drawing boundaries and measuring Social Innovation

## Discussion and Conclusions

A welfare originally designed to guarantee social rights to citizens by the state and managed through indebtedness and privatization may not be any more sustainable in the future considering the current demographic trends in Western societies. The public sector is no longer able on its own to respond to complex needs for social protection and is increasingly resorting to collaborations and partnerships with non-public actors that are embedded in the territories and that know people's needs closely (e.g., Rees et al., 2022). Both the crisis of capitalism and the crisis of the welfare state call for a better use of the different types of resources available in the whole economic system with a view to true sustainability capable of ensuring that the needs of current and future generations are met. A new vision could view welfare as an instrument for support, insurance and human flourishing which is aimed at an individual citizen who is in a relationship with a community of reference (circle in the words of Cottam, 2016), and no longer considered merely as an individual client entitled to a set of individualized benefits. However, this vision could seem a kind of romantic policy utopia never achievable or rather a democratic heterotopia where multiple experimental places are offered to publics and stakeholders for inclusive social innovation to deal with the welfare problems and social needs emerging from circumstances.

Against this backdrop, this essay tried to deal with the issue of institutionalizing social innovation as a bottom-up strategic response to the welfare state crisis by taking the perspective of inclusive social innovation as a complementary and pragmatic democratic exercise characterized by working with key publics and stakeholders to develop deliberation, learning and socially innovative capacity. Moreover, by focusing primarily on institutions that may result from interactions between the state and civil society actors, the paper mobilized the concepts of utopias and heterotopias from Foucault to promote a cognitive shift in the way the issue of institutionalizing social innovation is observed, interpreted, and implemented by public purpose organizations. The originality of our perspective aims to be the understanding of the public purpose organization (for example municipalities) as potentially with resources with a transformative capacity for inclusive social innovation and in linking more clearly social innovation to democratic, rather than market logics, with implications on the way social innovation is assessed and scaled.

Specifically, we have highlighted some obstacles to institutionalizing social innovation and some key governance mechanisms that can be activated either by public and/or social purpose organizations to foster that endeavour. The public sector has indeed an important democratic role in organizing the network society, even if it means changing its own paradigm, such as for example combining commons-based peer production with the neo-Weberian state (Kostakis, 2011). In this respect, we believe it will be crucial to study how the municipality, as the democratic pivot of place-based governance, can design, convene, and facilitate experimental places for social innovation focused on problem solving and opportunities generating through deliberation, sedimentation of experiences, design and re-imagination. Similarly, social purpose organizations have a key role for social reintermediation (e.g., Elstub, 2006; Pestoff, 2012) to participate in those arenas or to devise even counter-governance arenas to make pressure on existing public sector institutions to favour experimental places for inclusive social innovation.

The link between social innovation as emancipatory deliberation, dialogue and experimentation by publics and stakeholders connects with the development and practice of socio-political capability and participation (Moulaert et al., 2005), thus understanding inclusive social innovation as an antecedent for societal resilience and as an advanced practice of participatory democracy and of citizenship blending political and economic values. Clearly, there are risks of capture by existing institutions, power dynamics related to delegating power to decide from traditional democratic actors as well as issues regarding ‘the endowment of social capital available for creating a space for collective reflection (Tello-Rozas, 2016, p. 81).’

However, the main idea is that social innovation can become a political force for manufacturing new democratic institutions and for reforming the welfare state and the capitalistic order through digitally connected bottom-up social innovation initiatives (e.g., Kostakis, 2011). We believe that this civic culture of social innovation could help to inform existing experimentation with radical democratic community building around the world, opening up the possibility for digital and place-based networking and civic activism inspired by global human advancement and by a new humanism (e.g., Tang, 2019). The institutionalization either in public and/or social purpose organization of everyday practices and opportunities for inclusive social innovation may serve to ‘symbolize what is possible, and what not, within the limit of the politico-economic context in which they operate’ (Mazzei et al., 2021, p. 1638).

Institutionalizing heterotopia experimental places in public and social purpose organizations for inclusive social innovation as much as existing theatres, prisons, gardens, and museums do exist and function in our cities, villages and communities can ultimately promote “human flourishing and democratic engagement beyond instrumentalism”(Ansell, 2011). The question is bringing in through inclusive social innovation more experimental places as a deliberative and civic alternative to elitist conception of democracy, transforming unorganized groups of citizens and stakeholders into active ‘publics’ providing a web of support for dealing with increasingly emerging and evolving welfare needs. This is not a matter of utopias, but of designing and experimenting these possibilities as heterotopias within existing institutions, something that is a political choice to be done (or not) by public and social purpose organizations.

## Footnotes

1 According to the World Health Organization, between 2015 and 2050 the proportion of the world's population over 60 years will nearly double from 12% to 22%. Source: https://www.who.int/news-room/fact-sheets/detail/ageing-and-health, accessed on 5th August 2022.

2 The TRANSIT research project was co-founded by the European Commission and ran for four years, from January 2014 until December 2017, and was coordinated by DRIFT (Erasmus University Rotterdam—The Netherlands) and involved 12 universities and research institutes from across Europe and Latin America.

## References

[CR1] Agostini MR (2017). An overview on social innovation research: Guiding future studies. Brazilian Business Review (portuguese Edition).

[CR2] Alvesson M, Spicer A (2012). Critical leadership studies: The case for critical performativity. Human Relations.

[CR3] Ansell CK (2011). Pragmatist democracy: Evolutionary learning as public philosophy.

[CR4] Ansell C, Miura S (2020). Can the power of platforms be harnessed for governance?. Public Administration.

[CR5] Ansell C, Sørensen E, Torfing J (2021). The COVID-19 pandemic as a game changer for public administration and leadership? The need for robust governance responses to turbulent problems. Public Management Review.

[CR6] Austin MJ (2003). The changing relationship between nonprofit organizations and public social service agencies in the era of welfare reform. Nonprofit and Voluntary Sector Quarterly.

[CR7] Avelino F, Wittmayer JM, Pel B, Weaver P, Dumitru A, Haxeltine A, Kemp R, Jørgensen MS, Bauler T, Ruijsink S, O’Riordan T (2019). Transformative social innovation and (dis)empowerment. Technological Forecasting and Social Change.

[CR8] Ayob N, Teasdale S, Fagan K (2016). How social innovation “Came to Be”: Tracing the evolution of a contested concept. Journal of Social Policy.

[CR9] Baglioni S (2017). A remedy for all sins? Introducing a special issue on Social Enterprise and Welfare Regimes in Europe. VOLUNTAS: International Journal of Voluntary and Nonprofit Organizations.

[CR10] Baptista N, Pereira J, Moreira AC, Matos ND (2019). Exploring the meaning of social innovation: A categorisation scheme based on the level of policy intervention, profit orientation and geographical scale. Innovation: the European Journal of Social Science Research.

[CR11] Battilana J, Casciaro T (2021). Power, for All: How it really works and why it's everyone's business.

[CR12] Bock BB (2012). Social innovation and sustainability; how to disentangle the buzzword and its application in the field of agriculture and rural development. Studies in Agricultural Economics.

[CR13] Borzaga C, Bodini R (2014). What to make of social innovation? Towards a framework for policy development. Social Policy and Society.

[CR14] Brandsen T, Cattacin S, Evers A, Zimmer A (2016). Social innovations in the urban context.

[CR15] Brandsen T, Trommel W, Verschuere B (2017). The state and the reconstruction of civil society. International Review of Administrative Sciences.

[CR16] Bryson JM, Crosby BC, Bloomberg L (2014). Public value governance: Moving beyond traditional public administration and the new public management. Public Administration Review.

[CR17] Busacca M (2013). Oltre la retorica della Social Innovation. Impresa Sociale.

[CR18] Cajaiba-Santana G (2014). Social innovation: Moving the field forward—A conceptual framework. Technological Forecasting and Social Change.

[CR19] Carayannis EG, Grigoroudis E, Campbell DF, Meissner D, Stamati D (2018). The ecosystem as helix: An exploratory theory-building study of regional co-opetitive entrepreneurial ecosystems as Quadruple/Quintuple Helix Innovation Models. R&D Management.

[CR20] Carboni JL, Dickey T, Moulton S, O’keefe S, O’leary R, Piotrowski SJ, Sandfort J (2019). Start with the problem: Establishing research relevance with integrative public administration. Perspectives on Public Management and Governance.

[CR21] Chesbrough H, Di Minin A (2014). Open social innovation. New Frontiers in Open Innovation.

[CR24] Cottam, H. (2016). Social Services Are Broken. How We Can Fix Them. TED Talks, available at https://www.youtube.com/watch?v=Mr8nvXvl-y8&t=842s. Accessed 30 June 2022.

[CR25] Coule TM, Dodge J, Eikenberry AM (2022). Toward a typology of critical nonprofit studies: A literature review. Nonprofit and Voluntary Sector Quarterly.

[CR26] Dekker P, Evers A (2009). Civicness and the third sector: Introduction. VOLUNTAS: International Journal of Voluntary and Nonprofit Organizations.

[CR27] Dey P, Teasdale S (2016). The tactical mimicry of social enterprise strategies: Acting ‘as if’ in the everyday life of third sector organizations. Organization.

[CR29] Domanski D, Howaldt J, Kaletka C (2020). A comprehensive concept of social innovation and its implications for the local context—on the growing importance of social innovation ecosystems and infrastructures. European Planning Studies.

[CR30] Dorf MC, Sabel CF (1998). A constitution of democratic experimentalism. Columbia Law Review.

[CR31] Dyck, B. V., & den Broeck, P. V. (2013). Social innovation: A territorial process: Collective Action, Social Learning and Transdisciplinary Research. In *The international handbook on social innovation* (pp. 131–141). Edward Elgar Publishing. http://www.elgaronline.com/view/9781849809986.00021.xml

[CR32] Elstub S (2006). Towards an inclusive social policy for the UK: The need for democratic deliberation in voluntary and community associations. VOLUNTAS: International Journal of Voluntary and Nonprofit Organizations.

[CR33] Ewert B, Evers A (2014). Blueprints for the future of welfare provision? Shared features of service innovations across Europe. Social Policy and Society.

[CR34] Friant C, Vermeulen WJV, Salomone R (2020). A typology of circular economy discourses: navigating the diverse visions of a contested paradigm. Resources, Conservation and Recycling.

[CR35] Fung A (2002). Creating deliberative publics: Governance after devolution and democratic centralism. The Good Society.

[CR36] Garcia-Orosa B, Pérez-Seijo S (2020). The use of 360 video by international humanitarian aid organizations to spread social messages and increase engagement. VOLUNTAS: International Journal of Voluntary and Nonprofit Organizations.

[CR37] George G, Howard-Grenville J, Joshi A, Tihanyi L (2016). Understanding and tackling societal grand challenges through management research. Academy of Management Journal.

[CR38] Gruber TR (1995). Toward principles for the design of ontologies used for knowledge sharing?. International Journal of Human-Computer Studies.

[CR39] Hartley J (2005). Innovation in governance and public services: Past and present. Public Money and Management.

[CR40] Henriksen LS, Rathgeb Smith S, Zimmer A (2012). At the eve of convergence?. Transformations of Social Service Provision in Denmark, Germany and the United States, Voluntas.

[CR41] Henry M, Schraven D, Bocken N, Frenken K, Hekkert M, Kirchherr J (2021). ‘The Battle of the Buzzwords: A comparative review of the circular economy and the sharing economy concepts. Environmental Innovation and Societal Transitions.

[CR42] Hjelmar U (2021). The institutionalization of public sector innovation. Public Management Review.

[CR43] IFEL/ANCI-Cariplo (2022) Valore Comune. Un Patto Generativo di Comunita’ attraverso azioni di sistema participate per lo sviluppo dei territori.

[CR44] Jensen PH, Fersch B (2019). Institutional entrepreneurs and social innovation in Danish Senior Care. Administration & Society.

[CR45] Källström L, Mauro S, Sancino A, Grossi G (2021). The governance games of citizens and stakeholders’ engagement: Longitudinal narratives. Local Government Studies.

[CR46] Kania J, Hanleybrown F, Splansky Juster J (2014). Essential mindset shifts for collective impact. Stanford Social Innovation Review.

[CR101] Klijn, E. H., & Koppenjan, J. (2016). *Governance networks in the public sector*. Routledge.

[CR47] Kostakis V (2011). Commons-based peer production and the Neo-Weberian State: Synergies and inter dependencies. Administrative Culture.

[CR48] Kronsell A, Mukhtar-Landgren D (2018). Experimental governance: The role of municipalities in urban living labs. European Planning Studies.

[CR49] Lachapelle MD (2021). Emancipatory social innovation: Within and beyond the innovative society. VOLUNTAS: International Journal of Voluntary and Nonprofit Organizations.

[CR50] Lawrence TB, Hardy C, Phillips N (2002). Institutional effects of interorganizational collaboration: The emergence of proto-institutions. Academy of Management Journal.

[CR51] Lenz T, Shier ML (2021). Supporting transformational social innovation through nonprofit and local government relations: A scoping literature review. Human Service Organizations: Management, Leadership & Governance.

[CR103] Levitas R (1979). Sociology and utopia. Sociology.

[CR53] Macmillan R (2020). Somewhere over the rainbow—third sector research in and beyond coronavirus. Voluntary Sector Review.

[CR54] Mahoney J, Thelen K, Mahoney J, Thelen K (2010). A theory of gradual institutional change. Explaining institutional change: Ambiguity, agency and power.

[CR55] Mair J, Gegenhuber T (2021). Open social innovation. Stanford Social Innovation Review.

[CR56] Mair J, Kindt J, Mena S (2023). The emerging field of political innovation. Stanford Social Innovation Review.

[CR57] Marques P, Morgan K, Richardson R (2018). Social innovation in question: The theoretical and practical implications of a contested concept. Environment and Planning C: Politics and Space.

[CR58] Mazzei M (2017). Understanding difference: The importance of ‘place’in the shaping of local social economies. VOLUNTAS: International Journal of Voluntary and Nonprofit Organizations.

[CR59] Mazzei M, Montgomery T, Dey P (2021). 'Utopia'failed? Social enterprise, everyday practices and the closure of neoliberalism. Environment and Planning C: Politics and Space.

[CR60] Mazzucato M (2021). Mission economy: A moonshot guide to changing capitalism.

[CR61] Meijer AJ, Lips M, Chen K (2019). Open governance: A new paradigm for understanding urban governance in an information age. Frontiers in Sustainable Cities.

[CR62] Mihci H (2020). Is measuring social innovation a mission impossible?. Innovation: the European Journal of Social Science Research.

[CR63] Misuraca, G., Pasi, G., & Viscusi, G. (2018). Social innovation and resilience: Exploring the dynamics and impacts on the digital transformation of governance & society. In *Proceedings of the 11th international conference on theory and practice of electronic governance* (pp. 91–100). 10.1145/3209415.3209488

[CR64] Montagut, T., Vilà, G., & Riutort, S. (2016). Barcelona: A Citizen’s Agreement for an Inclusive City. Social Innovations in the Urban Context, (pp. 273–279).

[CR65] Montgomery T (2016). Are social innovation paradigms incommensurable?. Voluntas: International Journal of Voluntary and Nonprofit Organizations.

[CR104] Moulaert, F., Hillier, J., & Vicari Haddock, S. (Eds.). (2009). *Social innovation and territorial development*. Ashgate Publishing Ltd.

[CR67] Moulaert F, MacCallum D (2019). Social Innovation.

[CR68] Moulaert F, Martinelli F, Swyngedouw E, Gonzalez S (2005). Towards alternative model (s) of local innovation. Urban Studies.

[CR66] Moulaert, F., Mehmood, A., MacCallum, D., & Leubolt, B. (2017, September 1). *Social innovation as a trigger for transformations*—*The role of research* [*Monograph*]. Publications Office of the European Union. 10.2777/679791

[CR69] Mulgan, G., Tucker, S., Ali, R., & Sanders, B. (2007). Social Innovation: What it is, why it matters, how it can be accelerated. London: University of Oxford, Young Foundation. Available at: https://youngfoundation.org/wp-content/uploads/2012/10/Social-Innovation-what-it-is-why-it-matters-how-it-can-be-accelerated-March-2007.pdf. Accessed 19 May 2023.

[CR70] Nicholls A, Simon J, Gabriel M (2015). New Frontiers in Social Innovation Research.

[CR71] Parthasarathy B, Dey S, Gupta P (2021). Overcoming wicked problems and institutional voids for social innovation: University-NGO partnerships in the Global South. Technological Forecasting and Social Change.

[CR72] Pel, B., & Bauler, T. (2014). The institutionalization of social innovation: Between transformation and capture. TRANSIT working paper 2 (pp. 2–1).

[CR73] Pel, B., Wittmayer, J., Avelino, F. & Bauler, T. (2019). Paradoxes of Transformative Social Innovation: From Critical Awareness towards Strategies of Inquiry, presented at Research colloquium Critical and Interpretive Public Administration (CIPA) Nijmegen (NL), June 21st 2019, available at https://scholar.google.com/citations?view_op=view_citation&hl=it&user=52Yhu0oAAAAJ&sortby=pubdate&citation_for_view=52Yhu0oAAAAJ:rTD5ala9j4wC. Accessed on 28th June 2022.

[CR74] Pel B, Haxeltine A, Avelino F, Dumitru A, Kemp R, Bauler T, Kunze I, Jørgensen MS (2020). Towards a theory of transformative social innovation: A relational framework and 12 propositions. Research Policy.

[CR75] Pestoff V (2012). Co-production and third sector social services in Europe: Some concepts and evidence. VOLUNTAS: International Journal of Voluntary and Nonprofit Organizations.

[CR76] Phillips W, Lee H, Ghobadian A, O’Regan N, James P (2015). Social innovation and social entrepreneurship: A systematic review. Group & Organization Management.

[CR77] Phills JA, Deiglmeier K, Miller DT (2008). Rediscovering social innovation. Stanford Social Innovation Review.

[CR78] Rees J, Sancino A, Jacklin-Jarvis C, Pagani M (2022). ‘You can’t Google everything’: The voluntary sector and the leadership of communities of place. Leadership.

[CR105] Roy, M. J., Donaldson, C., Baker, R., & Kerr, S. (2014). The potential of social enterprise to enhance health and well-being: A model and systematic review. *Social Science & Medicine*, *123*, 182–193. 10.1016/j.socscimed.2014.07.031 25037852

[CR80] Ruddat C, Schönauer A-L (2014). New players on crowded playing fields: The institutional embeddedness of social innovation in Germany. Social Policy and Society.

[CR81] Sancino, A. (2022). *Public Value Co-Creation*—*A Multi-Actor & Multi-Sector Perspective*. Emerald Publishing. https://books.emeraldinsight.com/page/detail/public-value-co-creation/?k=9781803829623

[CR82] Sancino A (2016). The meta co-production of community outcomes: Towards a citizens’ capabilities approach. Voluntas: International Journal of Voluntary and Nonprofit Organizations.

[CR83] Sancino A, Garavaglia C, Sicilia M, Braga A (2021). New development: Covid-19 and its publics—Implications for strategic management and democracy. Public Money & Management.

[CR84] Setälä M (2017). Connecting deliberative mini-publics to representative decision making. European Journal of Political Research.

[CR85] Skelcher C, Sullivan H, Jeffares S (2013). Hybrid governance in European cities: Neighbourhood, migration and democracy.

[CR86] Solis-Navarrete JA, Bucio-Mendoza S, Paneque-Gálvez J (2021). What is not social innovation. Technological Forecasting and Social Change.

[CR87] Sørensen E (2006). Metagovernance: The changing role of politicians in processes of democratic governance. The American Review of Public Administration.

[CR88] Sørensen E, Torfing J (2019). Designing institutional platforms and arenas for interactive political leadership. Public Management Review.

[CR89] Tang, A. (2019). Digital social innovation to empower democracy. TED x Vitoria Gasteiz, 8th May 2019, available at https://www.youtube.com/watch?v=LscTx6DHh9I. Accessed 30 June 2022.

[CR90] Teasdale S (2012). Negotiating tensions: How do social enterprises in the homelessness field balance social and commercial considerations?. Housing Studies.

[CR91] Tello-Rozas S (2016). Inclusive innovations through social and solidarity economy initiatives: A process analysis of a peruvian case study. VOLUNTAS: International Journal of Voluntary and Nonprofit Organizations.

[CR92] Torfing J, Sørensen E, Røiseland A (2019). Transforming the public sector into an arena for co-creation: Barriers, drivers, benefits, and ways forward. Administration & Society.

[CR93] Unceta A, Castro-Spila J, García Fronti J (2016). Social innovation indicators. Innovation: the European Journal of Social Science Research.

[CR94] Van Wijk J, Zietsma C, Dorado S, De Bakker FG, Martí I (2019). Social innovation: Integrating micro, meso, and macro level insights from institutional theory. Business & Society.

[CR95] Vidler A, Foucault M, Johnston P (2014). Heterotopias. AA Files.

[CR96] Voorberg WH, Bekkers VJJM, Tummers LG (2015). A systematic review of co-creation and co-production: Embarking on the social innovation journey. Public Management Review.

[CR102] Westley F, Antadze N, Riddell DJ, Robinson K, Geobey S (2014). Five configurations for scaling up social innovation: Case examples of nonprofit organizations from Canada. The Journal of Applied Behavioral Science.

[CR97] Westley F, McGowan K (2017). The evolution of social innovation: Building resilience through transitions.

[CR98] Zanoni P, Contu A, Healy S, Mir R (2017). Post-capitalistic politics in the making: The imaginary and praxis of alternative economies. Organization.

[CR99] Zivkovic S (2018). Systemic innovation labs: A lab for wicked problems. Social Enterprise Journal.

[CR100] Zuckerman SJ (2020). “Why can’t this work here?”: Social innovation and collective impact in a micropolitan community. Community Development.

